# A Measure of the Signal-to-Noise Ratio of Microarray Samples and Studies Using Gene Correlations

**DOI:** 10.1371/journal.pone.0051013

**Published:** 2012-12-12

**Authors:** David Venet, Vincent Detours, Hugues Bersini

**Affiliations:** 1 IRIDIA (Institut de Recherches Interdisciplinaires et de Développements en Intelligence Artificielle), Université Libre de Bruxelles (ULB), Brussels, Belgium; 2 IRIBHM (Institut de Recherche Interdisciplinaire en Biologie Humaine et Moléculaire) & WELBIO (Walloon Excellence in Lifesciences and Biotechnology), Université Libre de Bruxelles (ULB), Brussels, Belgium; University of Iowa, United States of America

## Abstract

**Background:**

The quality of gene expression data can vary dramatically from platform to platform, study to study, and sample to sample. As reliable statistical analysis rests on reliable data, determining such quality is of the utmost importance. Quality measures to spot problematic samples exist, but they are platform-specific, and cannot be used to compare studies.

**Results:**

As a proxy for quality, we propose a signal-to-noise ratio for microarray data, the “Signal-to-Noise Applied to Gene Expression Experiments”, or SNAGEE. SNAGEE is based on the consistency of gene-gene correlations. We applied SNAGEE to a compendium of 80 large datasets on 37 platforms, for a total of 24,380 samples, and assessed the signal-to-noise ratio of studies and samples. This allowed us to discover serious issues with three studies. We show that signal-to-noise ratios of both studies and samples are linked to the statistical significance of the biological results.

**Conclusions:**

We showed that SNAGEE is an effective way to measure data quality for most types of gene expression studies, and that it often outperforms existing techniques. Furthermore, SNAGEE is platform-independent and does not require raw data files. The SNAGEE R package is available in BioConductor.

## Introduction

High-throughput gene expression data have been generated by microarrays for over ten years. The technology has, however, remained plagued with quality issues, to the point that a consortium (MAQC [1], [2]) was created to evaluate the reproducibility of those data. The conclusions of the consortium were reassuring, but this is of little help to the researcher who has to deal with a given data set, whose quality may be very different from MAQC. This is particularly problematic for data found on public repositories, as it is difficult to have first-hand information on a given study or sample.

While quality control metrics are available, they are platform-specific, and are meant to compare the relative quality of samples. There are currently no methods to compare different studies or different platforms. A number of R packages implement those quality metrics: Affyexpress, simpleaffy [3], and yaqcaffy for Affymetrix arrays, arrayQuality for spotted arrays and beadarray [4] for Illumina BeadArray. Once metrics have been calculated, outliers must be determined either visually using the displays offered by those packages, or automatically using MDQC [5] or arrayMvout [6]. The metrics are not comparable from study to study or platform to platform. They are also not comparable if samples have been treated with different protocols in the same study, for instance Affymetrix 1-round and 2-round amplification. Also, some problems, like scratches on the slides, can be difficult to detect by array-wide quality measures. Finally, the identification of problematic samples is dependent on the metrics chosen. For instance, housekeeping genes are often among the quality metrics used, but it has been shown that housekeeping genes may be regulated [7].

A different approach was proposed [8], based on the consistency of probe intensities in Affymetrix slides. Two different metrics were defined: relative log expression (RLE) which tests whether the number of up-regulated genes approximately equal the number of down-regulated genes, and the normalized unscaled standard error (NUSE), which tests if the standard deviation of the probe intensities of a given slide compared to the mean of its study is higher than average. Both methods rate slides inside their studies, and are unable to rate individual arrays. To address this shortcoming, a method was recently developped: GNUSE [9]. It is similar to NUSE, but instead of comparing the array to other arrays in the same study, it uses an empirical distribution, determined from a large compendium of arrays. By counting the number of problematic samples in a study, GNUSE can be used to assign quality to studies. A potential issue with those methods is that they are dependent on the details of the experimental protocol, as small differences in the methods used can lead to large differences in some probe average intensities. Another potential issue for GNUSE is that the samples assessed must be similar to those in the compendium.

This shows that there is a need for a quality measure that is more motivated by the underlying biology. Quality is a vague term, we propose to define here data quality as the statistical strength of the biological conclusions the data support. This could be expressed as the amount of biological signal relative to the amount of noise, that is a measure of the biological signal-to-noise ratio (SNR). As microarray studies can be used to answer many biological questions, possibly unrelated to the ones treated in the original publication, the strength of the biological signal is not determined based on sample annotations, but directly using the values of the microarray experiments. Whether SNR is a reliable estimate of data quality, in the sense of well-made experiments, depends on the study type. The signal-to-noise ratio depends not only on the amount of noise, which could be seen as a direct measure of quality, but also on the amount of signal. For instance, a high-quality study comparing a cell line in two different conditions could have very little variability, and so a low SNR. However, we show in this paper that SNR is a good proxy of quality for studies that comprise a large number of diverse samples, like for instance large studies on cancer tissues, and can reliably be used to rate comparable studies. It can also be used to flag problematic samples inside a study.

It has been shown [10] that gene-gene correlations are not random, but that sets of genes are often found to be similarly correlated across different studies and biological conditions. This can be expressed by saying that the gene-gene correlation matrix has a certain distribution, with some genes likely to be correlated while others are not. We propose here to use the distribution of the gene correlations as the basis of an SNR measure for all studies and platforms. The distribution of the gene correlations is estimated by using a large number of studies and platforms. The SNR of a study is obtained by comparing its gene correlations to the expected gene correlations. The SNR of individual samples is assessed by observing the difference in the SNR of the study they are part of when they are removed. The sample SNR is a measure of the relative contribution by a sample to the signal and noise of its study, so it is not a ratio, but we still use the term signal-to-noise ratio as it conveys the idea behind the measure. Working with gene-gene correlations has many advantages compared to existing techniques: it is based on a biologically meaningful concept, it works across studies, protocols and platforms, it can be applied to both studies and samples, it is sensitive to probe misannotation, it does not require access to the raw files, and it is fully automated.

The use of gene correlations to assess gene reproducibility has already been explored [11], to determine which genes are reproducibly regulated across studies. The reproducibility of gene correlations across a large number of studies was assessed [10], and coexpression links that increased the reliability of gene function inferrences were detected. More recently, probe correlations were used [12] to improve the annotations of Affymetrix probes, and to remove misleading or uninformative ones. But, to the best of our knowledge, it has not been used yet to determine study and sample SNR in a systematic fashion.

The study SNR was calculated on all studies in our database. This allowed to single out several studies having serious issues. We also show that studies with lower SNR lead to less significant biological results. The SNR measure for samples was studied in several datasets. We show that first it properly flags problematic samples, and second that statistically less significant results are obtained on those samples.

## Methods

### Measure of studies signal-to-noise ratio

We estimate the SNR of a study using correlation of gene correlations. A study here is a set of hybridizations done as a group on one platform—if the data relative to a publication consists of more than one platform, then there are as many studies as there are platforms.

The expected matrix of gene-gene correlations 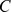 is estimated as the median of correlation matrices calculated across a large number of studies and platforms. The SNR of a study is obtained by comparing its gene correlations to 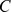. In practice, instead of directly comparing the correlations, their 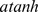 are taken, to allow correction for the number of samples (see below). As correlations rarely exceed 80%, this modification has little effect on the end result.

Specifically, data were put on a log scale and normalized, and multiple probes corresponding to the same gene were averaged (see the Data section for details). From the data matrix 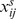 of study 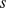, the Pearson correlation 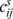 between genes 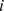 and 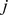 in study 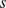 is calculated:
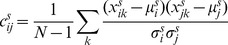
(1)where 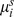 and 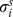 are the mean and standard deviation of gene 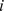 in study 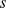. Let 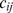 be the median of those correlations across all studies. The SNR 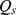 of study 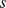 is the correlation between 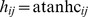 and 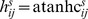, the diagonal excluded:
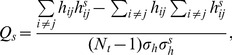
(2)where 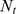 is the total number of gene-gene correlations, 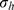 is the standard deviation of the gene-gene correlations for the median matrice, the diagonal excluded, and 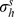 is the same for study 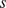.

#### Correction for the number of samples

The error on the measure of gene-gene correlations decreases for large studies. If the data are normally distributed, their 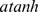 are normally distributed with a variance [13]

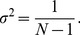
(3)Using this estimate of the variance of the gene-gene correlation, it is possible to disattenuate the correlation (2), that is, to calculate the correlation that would be obtained on an infinite number of samples [14]

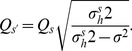
(4)


### Measure of signal-to-noise of samples inside their study

We estimate the SNR of a sample as the difference between the SNR of its study and the SNR of its study when the sample is removed. Removing a low SNR sample should increase the SNR of its study. The SNR of a study with a sample removed can be calculated efficiently since a sample has a simple additive effect on the mean, variance and covariance. We used a slightly modified SNR measure for this comparison:
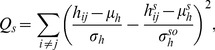
(5)where 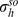 is the standard deviation of the original study, with all samples. Replacing 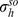 by 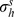 would make (5) equivalent to (2). The reason for this change is that some problematic samples have a very large effect on this standard deviation, which can be larger than the effect on the scalar product and fool the SNR measure.

The SNR 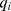 of sample 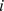 is the difference between the SNR of the complete study and the SNR of the study with that sample removed: 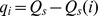. To make the results easier to understand, 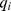 is normalized: 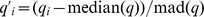, where 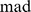 is the median absolute deviation over all the samples. This assigns a SNR to each sample, positive for better-than-average samples and negative for worse-than-average samples.

### Data

All calculations were done on the 2000 genes that were present among the most platforms in our database. More precisely, for each gene the number 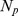 of platforms in which it appeared at least once was recorded. The genes were sorted in function of 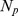, and the 2000 genes with the highest 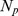 were kept.

Most datasets were downloaded from GEO [15], using the SeriesMatrix files (details about the datasets can be found in Table S1). No effort was made to ensure a specific distribution of tissue or study types, the data used reflects that of publicly available studies. All values were log-transformed if needed. The studies were normalized using the R function medpolish. Probes with more than 25% missing values were discarded. Probe annotations were either obtained using the annotation file from GEO, if available, or the mapping was done from the platform description file using the Gene and UniGene databases [16]. When multiple probes corresponded to a unique gene, their median was taken.

The correlations between genes were calculated for each platform and each study, giving as many correlation matrices as there are study/platform pairs, that is 101 in our case. The median of those matrices was taken, leading to a single 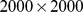 matrix of correlations.

### Validation

The relevance of our SNR measure was shown using methods detailed below.

#### Study SNR on NCI60

Four NCI60 studies were taken (GSE7947, GSE2003, GSE5949 and GSE5720), leading to a total of 9 study/platform pairs. We considered the 58 cell lines in common between those studies. SNR were not disattenuated as all data sets have the same number of samples. For each tissue type, the fraction of differentially expressed probes at 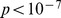 was calculated using a 2-sided T-test.

#### Sample SNR

SNAGEE was compared with simpleaffy, NUSE, RLE, GNUSE and MDQC. Simpleaffy is an R package that can be used to calculate quality metrics on an array. Those metrics are based on array-wide measures (*e.g.* background), control spots and the behaviour of specific genes. Bad quality arrays are found by MDQC as outliers in terms of those metrics.

To assess the biological relevance of the sample SNR, a 2-class classification was performed on a dataset, using a naive Bayes classifier, with Gaussian distributions and uninformative prior. Samples were classified in a leave-one-out fashion. The classifier gives a posterior probability for each assignement. Those probabilities were ranked and used as a proxy of the sample qualities, as bad samples should have higher lower posterior probabilities than average. The average of those ranks on the 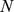 worst samples, as determined by our SNR criterion, MDQC or GNUSE, was then calculated.

## Results

Signal-to-noise ratios were assessed on a compendium of 80 studies on 37 platforms (Table S1), each comprising at least 50 samples and 200 genes, for a total of 101 study/platform pairs. All analyses were peformed on the 2000 genes that were present among the most platforms in our database (details are in Methods, section Data). We first assessed the study SNR, and then the individual sample SNR.

### Study SNR

#### Studies assigned a very low SNR have severe problems

We measured the SNR of all studies in our database. Three had a much lower SNR than expected. All of them had normalization problems.

The first study was GSE6768 [17], a study of breast cancers on an academic 2-colors platform, Swegene. The SNR of this study was very close to 0. Looking at the data in GEO, we realized that the values reported in the SeriesMatrix file did not fit the values of the individual samples. As the original GPR files were available, we reanalyzed the data using the R toolbox marray [18]. This increased the SNR to 0.15, which is about average for 2-color platforms.

The second study was GSE8833 [19], a study of cervical cancers on a 2-color platform, with dye swapping. The SNR of this study was very close to 0. The data downloaded from GEO contained 81% of values equal to zero, probably an artefact of the normalization used. As the GPR files were available, we were able to reanalyze the data. The renormalized data had a SNR of 0.06, an improvement on the original but still a low value. We checked if dye swapping impacted the SNR measure, by calculating separately the SNR on the hybridizations with the reference in channel 1, and on the dye-swapped hybridizations. The two SNR were 0.05 and 0.06, very close to the original. We also calculated the SNR of the dataset obtained by merging the dye-swapped samples. The resulting SNR, 0.07, was marginally better than the original SNR, presumably because some dye effects were removed.

The third study was GSE6532 [20], a large study on breast cancers using the U133A, U133B and U133+ Affymetrix chips. Its SNR on U133A and U133B seemed too low for those platforms. This was caused by the normalization, which had been done in three batches (Figure S1), as stated in the original publication. Since the original CEL files were available, we renormalized the data using the R function rma [21]. This increased the SNR from 0.23 to 0.38 on U133A and from 0.05 to 0.11 on U133B, which are average values for the platforms.

#### The SNR of a study is correlated with the statistical significance of biological findings

**Figure 1:**
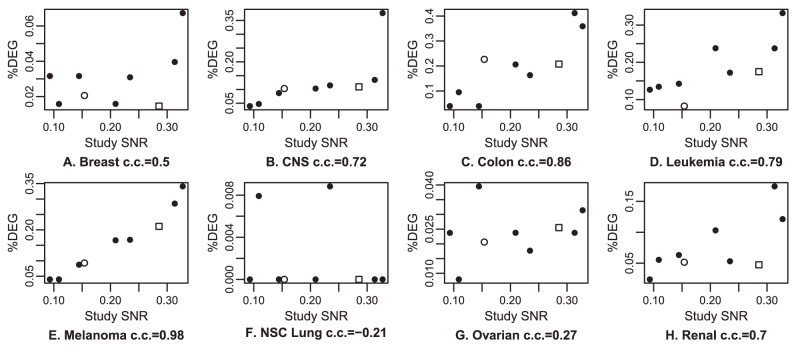
Link between statistical significance and study SNR in NCI60 datasets. Statistical significance is estimated as the fraction of differentially expressed genes (%DEG) between a cancer type and the other cancer types, each panel representing a different cancer type. Higher SNR leads to higher %DEG, as expected. 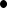 are on Affymetrix platforms (GSE5720 and GSE5949), 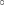 is on NCI dual channel (GSE2003) and 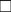 is on Stanford dual channel (GSE7947). c.c. are correlation coefficients.

We compared the statistical significances of different studies of the NCI60, a set of 60 well-characterized cancer cell lines. We used 4 studies, totaling 9 platforms. Taking the tissue of origin as the variable of interest, p-values were calculated for each gene and the fraction of differentially expressed genes (DEGs) was recorded (see Methods, section Validation on the NCI60). Studies with higher SNR should lead to a larger number of DEGs, since the same tests are performed on the same samples, measured on different platforms. We plotted for each cancer type the fraction of DEGs relative to the SNR of the studies (Figure 1). There is a clear correlation between SNR and the fraction of DEGs for most cancer types. In agreement with a previous study [22], out of the eight types represented by at least 3 samples, four (CNS, colon, leukemia, melanoma) gave a large number of DEGs, one an intermediate number (renal), two others (breast and ovarian) a small but significant number while very few genes were selected for lung. Much clearer results were obtained on types with a large fraction of DEGs. The correlation between the study SNR and the fraction of DEGs varied between 72% and 98% for the 4 types with a large number of DEGs. It remained positive for all other types but lung. So, SNR is predictive of the statistical significance that can be obtained from a study.

#### Random noise decreases both study SNR and statistical significance

**Figure 2:**
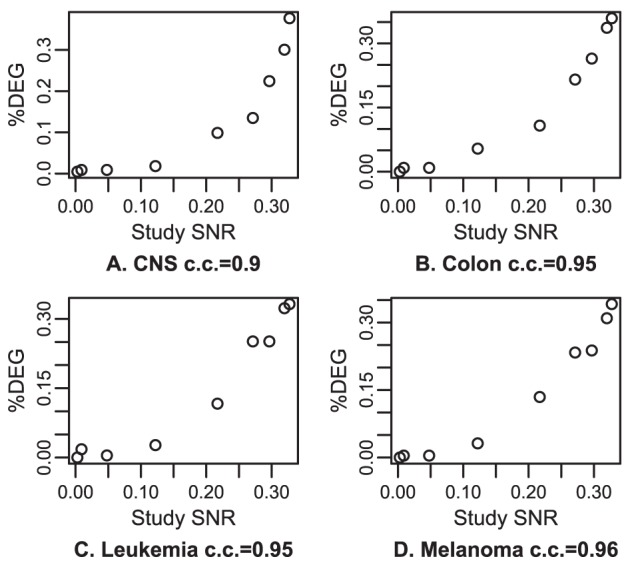
Effect of increasing levels of noise on study SNR and statistical significance. Statistical significance was estimated as the fraction of differentially expressed genes (%DEG). Noise was added to a study of the NCI60 on the U133A platform (GSE5720). SNR and %DEG were calculated on the modified study. Increasing noise lead to lower SNR and lower %DEG. c.c. are correlation coefficients, which underestimate the strength of the relations as those are not linear.

A specific study on the NCI60 cell lines was taken (GSE5720 on U133A—similar results were obtained with other datasets), and increasing amounts of Gaussian noise were added. This was done by fixing a level of noise 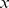, and adding values taken from a 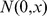 to the gene expression matrix. A number of studies were created with increasing noise level (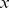). The resulting SNR and the resulting fraction of DEGs were compared. The correlation between the fraction of DEGs and the study SNR was well over 90% for the cancer types with a large number of DEGs (Figure 2), even though the relationship was not linear.

#### Biological outliers increase the signal-to-noise of a study

**Figure 3:**
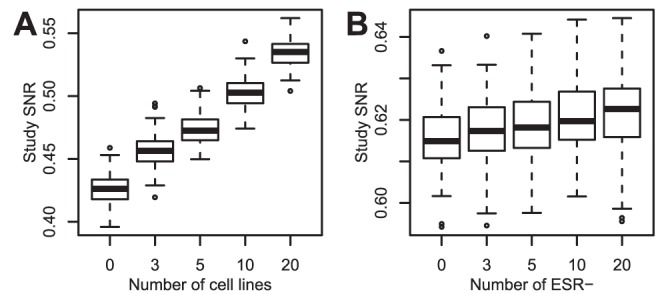
Effect of biological outliers on study SNR. The study SNRs were calculated in function of the number of biological outliers added to a study consiting of homogeneous samples. (**A**) Outliers are cell lines, original study consists of tumor and normal tissues. (**B**) Outliers are ESR− breast cancers, original study consists of ESR+ breast cancers.

The SNR is a measure of the amount of biological signal divided by the amount of noise. As such, it is increased by the presence of biological outliers. This stands in contrast with other quality measures, which search for outliers and so risk flagging biological outliers as low quality [5]. This was assessed using GSE7307, a study of normal and diseased tissues, plus cell lines. Cell lines are biologically very different from *in vivo* tissues [23]. We separated the data in two groups, one comprising all normal and diseased tissues (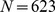), and the other comprising all the cell lines (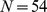). The SNR of studies consisting of 200 randomly drawn samples, a small number from group 2 and the rest from group 1, were calculated (Figure 3A). The addition of a small number of cell lines increased the study SNR.

We took a study of breast cancers (GSE6532 on U133A) as another illustration, with less striking outliers. An important feature of those cancers is the status of the estrogen receptor. An ESR− sample is an outlier in a study of only ESR+ samples. The SNR of studies consisting of 150 randomly drawn samples, a small number of ESR−, the rest being ESR+, was calculated (Figure 3B). The SNR slightly increased with the number of ESR− samples. The difference in SNR is significant with 10 ESR− samples (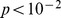) and 20 ESR− samples (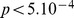).

#### Disattenuation makes SNR independent of the number of samples

**Figure 4:**
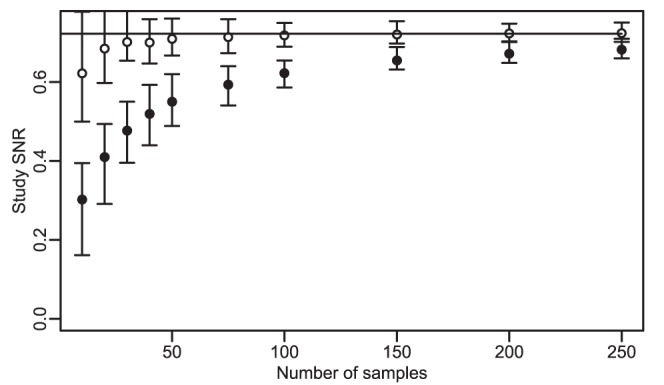
SNR in function of the number of samples: effect of disattenuation. Subsets of a very large dataset (expO) were created by randomly selecting a number of samples. The study SNRs of those subsets are shown in function of their size, with disattenuation (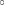) or without (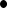). The error bars are the 95% confidence intervals determined by resampling. The horizontal line is the SNR of the whole data set.

SNR was calculated on random small subsets of the expO dataset (GSE2109, a dataset of 2149 cancer profiles), using disattenuation (4) or not. The results (Figure 4) show that disattenuation corrects to a large extent the effect of the study size on SNR.

#### Biological variability allows SNR measures on low signal studies

Experiments measuring smaller biological signals are assigned a lower SNR. However, the SNR remains meaningful if there is some biological variability in addition to the signal. For instance, GSE1322 [24] is a study of the effect of Japanese humor on diabetic patients. One would expect the biological signal to be faint, but the SNR of this study (0.24) was higher than average for the platform (Agilent 2-colors). This is due to the additional variability between the patients, which was not the signal of interest for the original publication.

#### Comparison with GNUSE

GNUSE allows to determine the quality of individual samples. By counting the number of such problematic samples, a study quality can be computed. We compared this study quality with the SNR determined by SNAGEE. Quite surprisingly, we found very little overlap between the two, albeit the five studies with 100% problematic samples, as determined by GNUSE, had indeed a slightly lower SNR than average (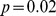, Mann-Whitney U-test).

A number of studies were given a low SNR while deemed of a good quality by GNUSE. Looking at those studies (with an SNR below 0.15: GSE9826, GSE8192 and GSE10063) we found that they were very targeted: they all consisted of a single cell line cultivated in a single medium, with different treatments. Such design, meant to highlight a single biological effect, leads to a low biological SNR.

A contrario, some studies were given a high SNR while GNUSE gave them a low quality. The authors of GNUSE could not pinpoint why some studies had a low quality. Looking at the list of studies that had over half bad samples from GNUSE but an SNR over 0.3, we found that half (6/12) used RNA amplification. This hints that the difference in the RNA preparation leads to sizeable differences in the raw probe intensities. SNAGEE, being based on gene correlation, is largely immune to those effects. Another study flagged by GNUSE but not SNAGEE is GSE9716, a study of tumor xenografts. It is likely that those tumors contain a mix of cells from mouse and human. Hybridization efficiency is expected to be very different between the two species. However, we found that gene correlations were largely conserved. We did not find a plausible explanation for the remaining 5 studies.

### SNR of the samples

#### Some problematic samples are similarly detected by SNAGEE, RLE, NUSE and GNUSE

The SNR assigned by SNAGEE to each sample was compared to the quality measure of RLE, NUSE, GNUSE as well as the metrics given by simpleaffy and MDQC. We first used the breast cancer dataset from MAQC-II (GSE20194). Table 1 shows a comparison between the different quality measures. In this case, good agreement was found between the main methods for the 10 worst slides, as determined by SNAGEE. We also used a study of 289 breast cancers on Affymetrix U133A (GSE4922 [25]). The sample SNR on U133A varied between −74 and +6. Table 2 shows a comparison between the simpleaffy metrics, and the quality measures. The two clearly problematic samples in this study were flagged similarly by all methods. There is also some agreement between the methods for other samples, although it is not perfect.

**Table 1:** Comparison of SNAGEE, MDQC, GNUSE and sample quality metrics on the breast cancer study from MAQC-II.

Sample	SNR	mdqc	gnuse	nuse	rle	%pres	scale	bioB	act5
GSM505497	−26	5	6	9	3	5	267	276	5
GSM505498	−25.3	4	3	4	4	2	264	271	9
GSM505495	−24.7	3	7	11	1	3	274	269	6
GSM505499	−24.1	2	5	8	2	4	273	263	4
GSM505492	−16.6	17	11	10	5	7	263	261	17
GSM505502	−15.9	13	9	12	7	8	268	259	10
GSM505500	−11.8	15	13	14	6	10	270	265	25
GSM505501	−11.2	10	12	17	9	11	266	260	1
GSM505491	−10.7	40	15	25	10	13	258	268	132
GSM505496	−8.8	11	8	7	8	9	277	277	15

The 10 worst samples as determined by SNAGEE are shown. *SNR* is the SNR from SNAGEE. *mdqc*, *gnuse*, *nuse* and *rle* are the rankings of the qualities obtained by MDQC, GNUSE, NUSE and RLE, 1 being the worse sample. The remaining values are the ranks of some Affymetrix quality metrics, from 1 to 278. Values close to 1 or 278 possibly indicate outliers.

**Table 2:** Comparison of SNAGEE, MDQC, GNUSE and sample quality metrics on a breast cancer study (GSE4922).

Sample	SNR	mdqc	gnuse	nuse	rle	%pres	scale	bioB	act5
GSM119943	−79.2	1	1	1	1	1	289	289	1
GSM119942	−78	2	2	2	2	2	287	288	2
GSM119963	−12.5	9	12	7	9	5	260	229	44
GSM119968	−10.4	25	9	12	73	8	238	243	167
GSM119945	−7.6	12	7	10	14	9	281	253	8
GSM119950	−7	5	11	8	6	4	284	286	4
GSM110704	−4.3	4	8	6	4	7	268	1	155
GSM119952	−3.6	3	4	3	8	3	288	287	89
GSM110820	−3.6	15	13	16	44	11	246	144	28
GSM110841	−3.1	7	10	9	3	6	286	284	25

The 10 worst samples as determined by SNAGEE are shown. *SNR* is the SNR from SNAGEE. *mdqc*, *gnuse*, *nuse* and *rle* are the rankings of the qualities obtained by MDQC, GNUSE, NUSE and RLE, 1 being the worse sample. The remaining values are the ranks of some Affymetrix quality metrics, from 1 to 289. Values close to 1 or 289 possibly indicate outliers.

#### Low SNR samples are harder to classify in breast cancer

Problematic samples can decrease the quality of the analysis performed. To verify that, we tried to classify samples with a naive Bayes classifier. Since bad quality samples should not fit the classification well, the posterior probabilities can be used as a proxy for sample qualities: bad quality samples should have a lower probability than average (see Methods). However, low probability samples are not necessarily of bad quality. There could be good reasons for the lack of fit to the classification—for instance, samples could belong to a rare cancer subtype. We checked here if bad quality samples (as determined by SNAGEE, MDQC, RLE, NUSE or GNUSE) had lower posterior probabilities than average.

**Figure 5:**
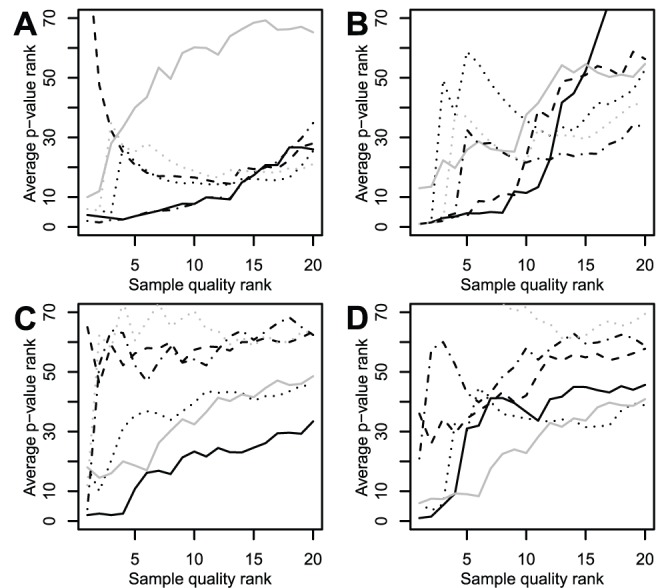
Comparison of quality metrics with Bayes classifier posterior probabilities. Samples were classified by a naive Bayes classifier. Bad quality samples should not fit the classification well, and have lower probabilities. The average of the ranks of the posterior probabilities of the 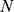 lowest quality samples is shown. Quality of the samples was determined with SNAGEE (solid line), MDQC (dashed line), GNUSE (dotted line), NUSE (grey dotted line) and RLE (dash-dot line). Classification was done on breast cancers according to ER status (**A**:GSE20194 and **B**:GSE4922); on muscle disease samples according to gender (**C**) or to the muscle disease type (**D**), one class vs. the rest. The solid grey line uses the samples assigned the highest SNR (and so likely to be biological outliers) by SNAGEE.

First, we took the breast cancer dataset from MAQC-II [2] (GSE20194), using all samples. This dataset was chosen because it has a few samples that were flagged as bad quality and discarded in the original analysis. We tried to classify the samples according to their ER status. We found that (Figure 5A) for a moderate number of problematic samples, SNAGEE was one of the best pronosticator of difficulty in classification on this dataset, on par with RLE. With a larger number of samples taken as problematic, all methods behave similarly. Interestingly, samples given a very good SNR by SNAGEE (shown in grey) were also more difficult to classify than average. This makes sense since those samples are likely to be biological outliers, that is, samples that do not fit well with the others but remain biologically plausible.

Second, a similar analysis was performed on another breast cancer dataset (GSE4922). Low SNR samples did not fit the classification well and had lower posterior probabilities (Figure 5B). Based on the figure, on this dataset SNAGEE and MDQC perform similarly, while the other methods were not as effective. Again, high SNR samples also did not fit the classification well.

#### SNAGEE outperforms MDQC in a muscle disease study

As another illustration, we calculated the qualities of the samples from a study of muscle diseases [26] on the U133A platform (GSE3307). We realized that MDQC assigned a low quality to all amyotrophic lateral sclerosis (ALS) samples. This was caused by the use of GAPDH probes in the quality metrics. GAPDH is commonly used as a housekeeping gene, but has been shown to be altered in mouse models of ALS [27]. So, GAPDH was removed for the calculations by MDQC. The qualities measured by MDQC and the SNR measured by SNAGEE had very little overlap. The significance of the samples with a low SNR was assessed with the same technique as for the breast cancer data, using gender as the variable of interest (Figure 5C). The worst four samples flagged by SNAGEE were among those harder to classify. Those flagged by MDQC, RLE and NUSE were no different than average. Surprisingly, GNUSE outperformed NUSE on this dataset. We then used the type of muscle disease as the variable of interest, and tried to classify each type vs. the rest. As statistics are not directly comparable for diseases having a different number of samples, and because the posterior probabilities can vary from group to group, less clear-cut results were expected. As shown Figure 5D, the first two samples flagged by SNAGEE and GNUSE were among those harder to classify, while those flagged by the other methods were no different than average. In this case the best predictor of the difficulty to classify was the inverse of SNAGEE, that is the high SNR samples were the hardest to classify.

#### Low SNR samples are harder to classify on the NCI60

In the analysis of the relationship between statistical significance and study SNR (Figure 1), statistical significance for a cancer type depended not only on the SNR of the study, but also on the relative SNR of the samples of that type compared to the rest of the samples. We assessed this on the dual-channel microarray studies. In GSE7947 (Stanford platform), one breast sample was given a low SNR, and the %DEG for that study on breast was indeed lower than expected. Another issue with this study is that a melanoma cell line has been annotated as a renal cell line (Figure S2), leading to a lower %DEG for the renal tissue. For GSE2003 (NCI platform), two leukemia samples were given a low SNR, and the %DEG for that study on leukemia was indeed lower than expected.

#### Biological outliers are assigned a higher SNR

**Figure 6:**
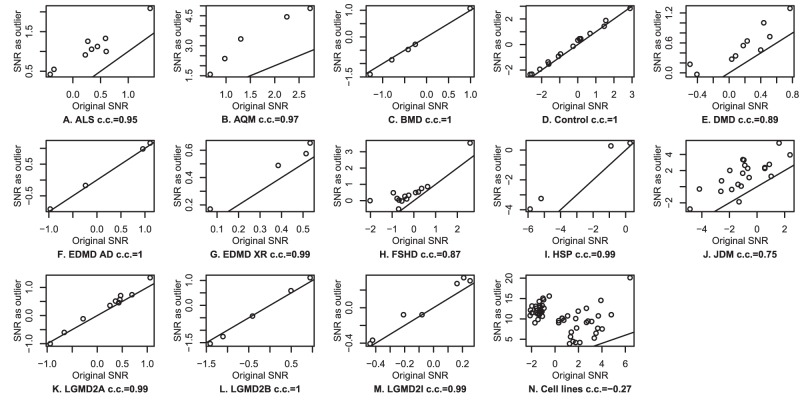
SNR of samples as biological outliers. The SNR of the samples within the complete study (x-axis) are compared with their SNR when removing all similar samples (y-axis). **A–M.** Muscle diseases, **N.** Cell lines vs. normal and diseased tissues.

We studied the behavior of SNAGEE on biological outliers using the study on muscle diseases (GSE3307). The SNR for each sample was first measured. Each disease was then taken in turn, and all samples from that disease were removed but one. That sample was then in the situation of being a biological outlier. The SNR of that sample was calculated. As can be seen Figure 6, for most diseases the SNR of the samples taken as biological outliers is higher than their SNR in the whole study. For some diseases (e.g. BMD or the LGMDs), the SNR were essentially identical. This is because those diseases are not outliers compared to the rest of the data—for instance, the 3 LGMDs are similar diseases with different genetic causes, so their gene expression profiles are similar.

For a more dramatic effect, we used the study of normal and diseased tissues, plus cell lines (GSE7307). The same analysis as with the muscle diseases was made, using the cell lines as the biological outliers. As shown Figure 6N, the SNR of the cell lines were much larger when measured as biological outliers.

## Discussion

We have shown on the NCI60 datasets that SNAGEE ranked the studies in a statistically relevant manner, in that more significant results were found on studies with a higher SNR. This was the case even though p-values were calculated at the probe, not the gene, level, and did not take gene annotations into account. We have also shown in a controlled setting that SNAGEE gives a direct measure of the added noise. This shows that the study SNR measure of SNAGEE is useful to compare studies with similar samples.

SNAGEE is a signal-to-noise ratio. As such, it increases if the signal—the variation in the data—is increased. This fits the concept that higher SNR studies hold more information, but must be kept in mind when comparing studies with different experimental contents. This is why we studied extensively the behavior of SNAGEE relative to biological outliers. SNAGEE gave them consistently a higher SNR than warranted. This means that the measure of SNR for a sample is not a measure of quality, but a measure of the added biological diversity brought by a sample to its study. A biologically plausible sample (a biological outlier) will be given a high SNR even if it has moderate technical issues, while an average sample with moderate technical issues will be given a low SNR. This is in contrast with results from MDQC, which gave a low quality to a category of biological outliers in the muscle disease study. SNAGEE also gave a higher SNR to complex studies, with different types of samples, than to more focused studies, all other things being equal.

A main limitation of SNAGEE is that it is dependent on the biological variation between samples. The signal-to-noise ratio at the core of SNAGEE is a proxy for quality only if there is some biological variablity between the samples. Very focused studies, for instance comparing only two cell lines, or one cell line treated with small stimuli under very controlled conditions, have often very low biological variability, and may be assigned a low SNR. In this case, the low SNR is telling of the experimental conditions and not of study quality. Similarly, studies with only few different conditions cannot be reliably assigned an SNR, since gene-gene correlations have to be calculated on a fair number of different samples. In particular, it was not possible to apply SNAGEE to MAQC, the benchmark for quality control of microarrays, as it consists only of two cell lines measured in many replicated ways. Typically, SNAGEE gave reasonable estimate of the quality of studies above 20 samples, unless those samples are replicated measures of a few well-controlled conditions, like MAQC.

The SNR given by SNAGEE is an estimate of the amount of biological information available in the gene expression measure of a study. A higher SNR does not necessarily means that a study is more adequate to answer a given biological question than another study. This depends largely on the samples measured and the quality of their annotations, which are beyond the scope of SNAGEE. For instance, in the case of cancer tissues, a study with carefully dissected samples could have less biological variability than another study with samples contaminated by adjacent tissues. The first study may be more informative about the state of the cancer, while the latter would have a higher SNR. Practically, however, for similar studies the variation in SNR is mostly due to the noise, not the signal, which explains why SNAGEE gave a reasonable estimate of study quality in the cases presented.

The study quality measure being dependent on the study type, and the natural variation between its samples, it should only be used to compare similar studies. The exception for this is studies where problems are suspected, as a very low SNR (below 5%) is telling of serious quality problems, and SNR much below those usual for the platform may be telling of systematic issues, like for instance the normalization by batches that was shown.

A strength of SNAGEE compared to available quality metrics is that it works across platforms. However, if the goal is to compare studies on the same platform, or to compare samples, the use of a platform-specific gene-gene correlation matrix could be an improvement. This would improve the accuracy of SNR determination by getting rid of the probe annotation problem. Since a large number of studies are necessary to reliably determine the gene-gene correlation matrix, this could only be done on popular platforms.

SNAGEE was compared to other methods for the detection of problematic samples. Some samples, among which the most problematic ones, were flagged similarly by all methods, while other samples were ranked differently. We used the posterior probability of a naive Bayes classifier to assess the validity of those rankings. On the examples shown, SNAGEE was shown to outperform the existing methods. It was shown in particular that all methods can fail if the experimental setup interferes with the quality metrics, as in the muscle disease where GAPDH is regulated for MDQC, or mRNA amplification for GNUSE, or very simplified experimental setup for SNAGEE. This highlights the danger of using only one method for quality control. Furthermore, those methods cannot be used blindly, and their limitations must be taken into account. Hence, the three methods are complementary on Affymetrix slides. SNAGEE is however the only one available across platforms.

We have shown that SNAGEE flags samples that are harder to classify and so are of bad quality. It could be reasoned that samples can be harder to classify because they are biological outliers, but as SNAGEE gives biological outliers a high SNR this is not the case. Samples flagged by SNAGEE, and other methods are not always the same, though, which further shows that the techniques are complementary.

It could come as a surprise that average gene-gene correlations allow for the determination of SNR, since those correlations are expected to vary from study to study. We believe it works because gene-gene correlations have a distribution which is meaningful for any study, being largely based on genes that belong to the main cellular processes (e.g. respiration, cell-cyle, proliferation). Biological variability, independantly of the experimental protocol, slightly up- or down-regulates those processes in every sample. This is sufficient to ensure the presence of gene correlations. Of course, some genes behave differently in each biological system, but we showed that this does not prevent the successful use of SNAGEE.

In conclusion, we have presented a new biology-motivated signal-to-noise ratio measure for studies and samples. It is the first signal-to-noise ratio measure for complete gene expression studies. It was shown to work for a wide range of platforms. It was also shown to be resilient to changes in the experimental design, like dye-swapping, and in the number of samples.

## Supporting Information

Figure S1 **Values of the two probes for gene CRH in study GSE6532 platform U133A, using (A) data from GEO or (B) renormalized data. A batch effect is present is (A), but not in (B).**
(PDF)

Figure S2 **Clustering of the NCI60 from GSE7947 (black), GSE5949 (dark green, prefaced by an X) and GSE5720 (green, prefaced by an O), using Ward linkage and correlation.** Cell lines duplicated between the three studies cluster together, with the exception of A498 (red) which clusters with LOXIMVI (blue) only in GSE7947.
(PDF)

Table S1 List of platform / study pairs. Platforms ID are from GEO. Studies of the form GSExyz were downloaded from GEO. Others were downloaded from SGDI or the author web site.
(XLSX)

## References

[1] Shi L, Reid LH, Jones WD, Shippy R, Warrington JA, et al (2006) The microarray quality control (maqc) project shows inter- and intraplatform reproducibility of gene expression measurements. Nat Biotechnol 24: 1151–1161. 16964229 10.1038/nbt1239 PMC3272078

[2] Shi L, Campbell G, Jones WD, Campagne F, Wen Z, et al (2010) The microarray quality control (maqc)-ii study of common practices for the development and validation of microarray-based predictive models. Nat Biotechnol 28: 827–38. 20676074 10.1038/nbt.1665 PMC3315840

[3] Wilson CL, Miller CJ (2005) Simpleaffy: a bioconductor package for affymetrix quality control and data analysis. Bioinformatics 21: 3683–3685. 16076888 10.1093/bioinformatics/bti605

[4] Dunning MJ, Smith ML, Ritchie ME, Tavare S (2007) beadarray: R classes and methods for illumina bead-based data. Bioinformatics 23: 2183–2184. 17586828 10.1093/bioinformatics/btm311

[5] Cohen Freue GV, Hollander Z, Shen E, Zamar RH, Balshaw R, et al (2007) Mdqc: a new quality assessment method for microarrays based on quality control reports. Bioinformatics 23: 3162–3169. 17933854 10.1093/bioinformatics/btm487

[6] Asare AL, Gao Z, Carey VJ, Wang R, Seyfert-Margolis V (2009) Power enhancement via multi-variate outlier testing with gene expression arrays. Bioinformatics 25: 48–53. 19015138 10.1093/bioinformatics/btn591 PMC2638936

[7] Savonet V, Maenhaut C, Miot F, Pirson I (1997) Pitfalls in the use of several “housekeeping” genes as standards for quantitation of mrna: the example of thyroid cells. Anal Biochem 247: 165–7. 9126387 10.1006/abio.1997.2055

[8] Bolstad BM, Collin F, Simpson KM, Irizarry RA, Speed TP (2004) Experimental design and low-level analysis of microarray data. Int Rev Neurobiol 60: 25–58. 15474586 10.1016/S0074-7742(04)60002-X

[9] McCall M, Murakami P, Lukk M, Huber W, Irizarry R (2011) Assessing affymetrix genechip microarray quality. BMC Bioinformatics 12: 137. 21548974 10.1186/1471-2105-12-137 PMC3097162

[10] Lee HK, Hsu AK, Sajdak J, Qin J, Pavlidis P (2004) Coexpression analysis of human genes across many microarray data sets. Genome Res 14: 1085–1094. 15173114 10.1101/gr.1910904 PMC419787

[11] Cope L, Zhong X, Garrett E, Parmigiani G (2004) Mergemaid: R tools for merging and cross-study validation of gene expression data. Statistical applications in genetics and molecular biology 3. 10.2202/1544-6115.1046 16646808

[12] Alvarez MJ, Sumazin P, Rajbhandari P, Califano A (2009) Correlating measurements across sam-ples improves accuracy of large-scale expression profile experiments. Genome Biol 10: R143. 20042104 10.1186/gb-2009-10-12-r143 PMC2812950

[13] Gayen AK (1951) The frequency distribution of the product-moment correlation coefficient in random samples of any size drawn from non-normal universes. Biometrika 38: 219–247. 14848124

[14] Spearman C (1987) The proof and measurement of association between two things. By C. Spearman, 1904. Am J Psychol 100: 441–471. 3322052

[15] Barrett T, Troup DB, Wilhite SE, Ledoux P, Rudnev D, et al (2007) Ncbi geo: mining tens of millions of expression profiles–database and tools update. Nucleic Acids Res 35: D760–5. 17099226 10.1093/nar/gkl887 PMC1669752

[16] Sayers EW, Barrett T, Benson DA, Bryant SH, Canese K, et al (2009) Database resources of the national center for biotechnology information. Nucleic Acids Res 37: D5–15. 18940862 10.1093/nar/gkn741 PMC2686545

[17] Jumppanen M, Gruvberger-Saal S, Kauraniemi P, Tanner M, Bendahl PO, et al (2007) Basallike phenotype is not associated with patient survival in estrogen-receptor-negative breast cancers. Breast Cancer Res 9: R16. 17263897 10.1186/bcr1649 PMC1851391

[18] Wang J, Nygaard V, Smith-Sorensen B, Hovig E, Myklebost O (2002) Marray: analysing single, replicated or reversed microarray experiments. Bioinformatics 18: 1139–1140. 12176840 10.1093/bioinformatics/18.8.1139

[19] Chao A, Wang TH, Lee YS, Hong JH, Tsai CN, et al (2008) Analysis of functional groups of differentially expressed genes in the peripheral blood of patients with cervical cancer undergoing concurrent chemoradiation treatment. Radiat Res 169: 76–86. 18159954 10.1667/RR1045.1

[20] Loi S, Haibe-Kains B, Desmedt C, Lallemand F, Tutt AM, et al (2007) Definition of clinically distinct molecular subtypes in estrogen receptor-positive breast carcinomas through genomic grade. J Clin Oncol 25: 1239–1246. 17401012 10.1200/JCO.2006.07.1522

[21] Irizarry RA, Hobbs B, Collin F, Beazer-Barclay YD, Antonellis KJ, et al (2003) Exploration, normalization, and summaries of high density oligonucleotide array probe level data. Biostatistics 4: 249–64. 12925520 10.1093/biostatistics/4.2.249

[22] Ross DT, Scherf U, Eisen MB, Perou CM, Rees C, et al (2000) Systematic variation in gene expression patterns in human cancer cell lines. Nat Genet 24: 227–35. 10700174 10.1038/73432

[23] van Staveren WCG, Solís DW, Delys L, Duprez L, Andry G, et al (2007) Human thyroid tumor cell lines derived from different tumor types present a common dedifferentiated phenotype. Cancer Res 67: 8113–20. 17804723 10.1158/0008-5472.CAN-06-4026

[24] Hayashi T, Urayama O, Kawai K, Hayashi K, Iwanaga S, et al (2006) Laughter regulates gene expression in patients with type 2 diabetes. Psychother Psychosom 75: 62–65. 16361876 10.1159/000089228

[25] Ivshina AV, George J, Senko O, Mow B, Putti TC, et al (2006) Genetic reclassification of histologic grade delineates new clinical subtypes of breast cancer. Cancer Res 66: 10292–10301. 17079448 10.1158/0008-5472.CAN-05-4414

[26] Bakay M, Wang Z, Melcon G, Schiltz L, Xuan J, et al (2006) Nuclear envelope dystrophies show a transcriptional fingerprint suggesting disruption of rb-myod pathways in muscle regeneration. Brain 129: 996–1013. 16478798 10.1093/brain/awl023

[27] Pierce A, Mirzaei H, Muller F, De Waal E, Taylor AB, et al (2008) Gapdh is conformationally and functionally altered in association with oxidative stress in mouse models of amyotrophic lateral sclerosis. J Mol Biol 382: 1195–210. 18706911 10.1016/j.jmb.2008.07.088 PMC6348903

