# Supplementary material for: A Measure of the Signal-to-Noise Ratio of Microarray Samples and Studies Using Gene Correlations
Source: PLoS One. 2012 Dec 12;7(12):e51013. doi: 10.1371/journal.pone.0051013 (PMC3520972; doi:10.1371/journal.pone.0051013)
Supplement: Figure S1 — Values of the two probes for gene CRH in study GSE6532 platform U133A, using (A) data from GEO or (B) renormalized data. A batch effect is present is (A), but not in (B). (PDF) [file pone.0051013.s001.pdf]

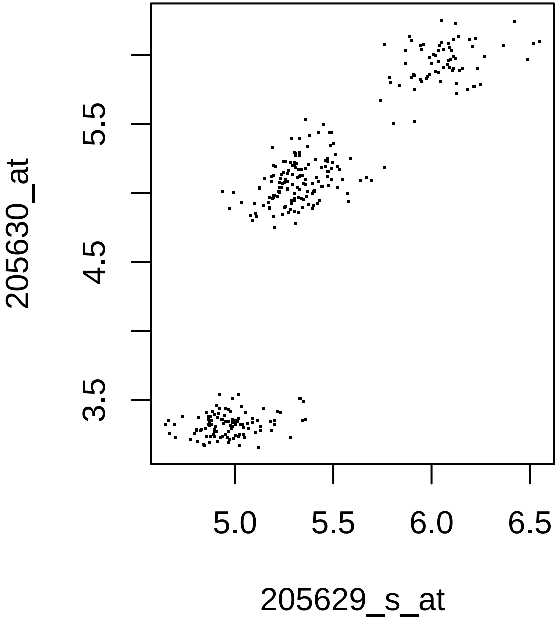

A

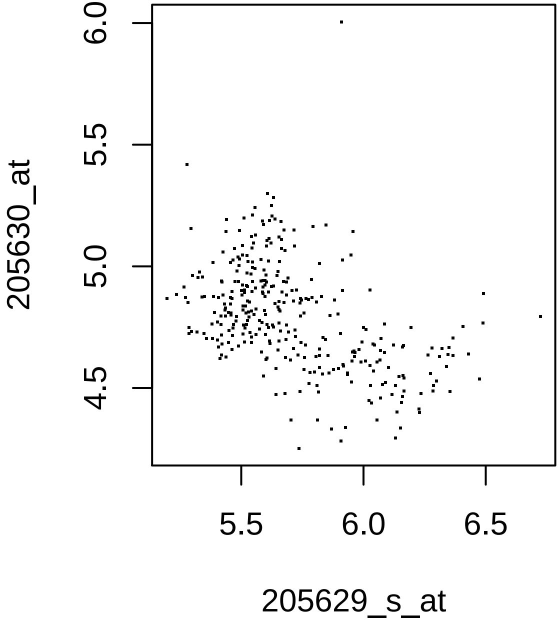

B

Figure S1. Values of the two probes for gene CRH in study GSE6532 platform U133A, using (A) data from GEO or (B) renormalized data.
A batch effect is present in (A), but not in (B).
