# Supplementary material for: A Measure of the Signal-to-Noise Ratio of Microarray Samples and Studies Using Gene Correlations
Source: PLoS One. 2012 Dec 12;7(12):e51013. doi: 10.1371/journal.pone.0051013 (PMC3520972; doi:10.1371/journal.pone.0051013)
Supplement: Figure S2 — Clustering of the NCI60 from GSE7947 (black), GSE5949 (dark green, prefaced by an X) and GSE5720 (green, prefaced by an O), using Ward linkage and correlation. Cell lines duplicated between the three studies cluster together, with the exception of A498 (red) which clusters with LOXIMVI (blue) only in GSE7947. (PDF) [file pone.0051013.s002.pdf]

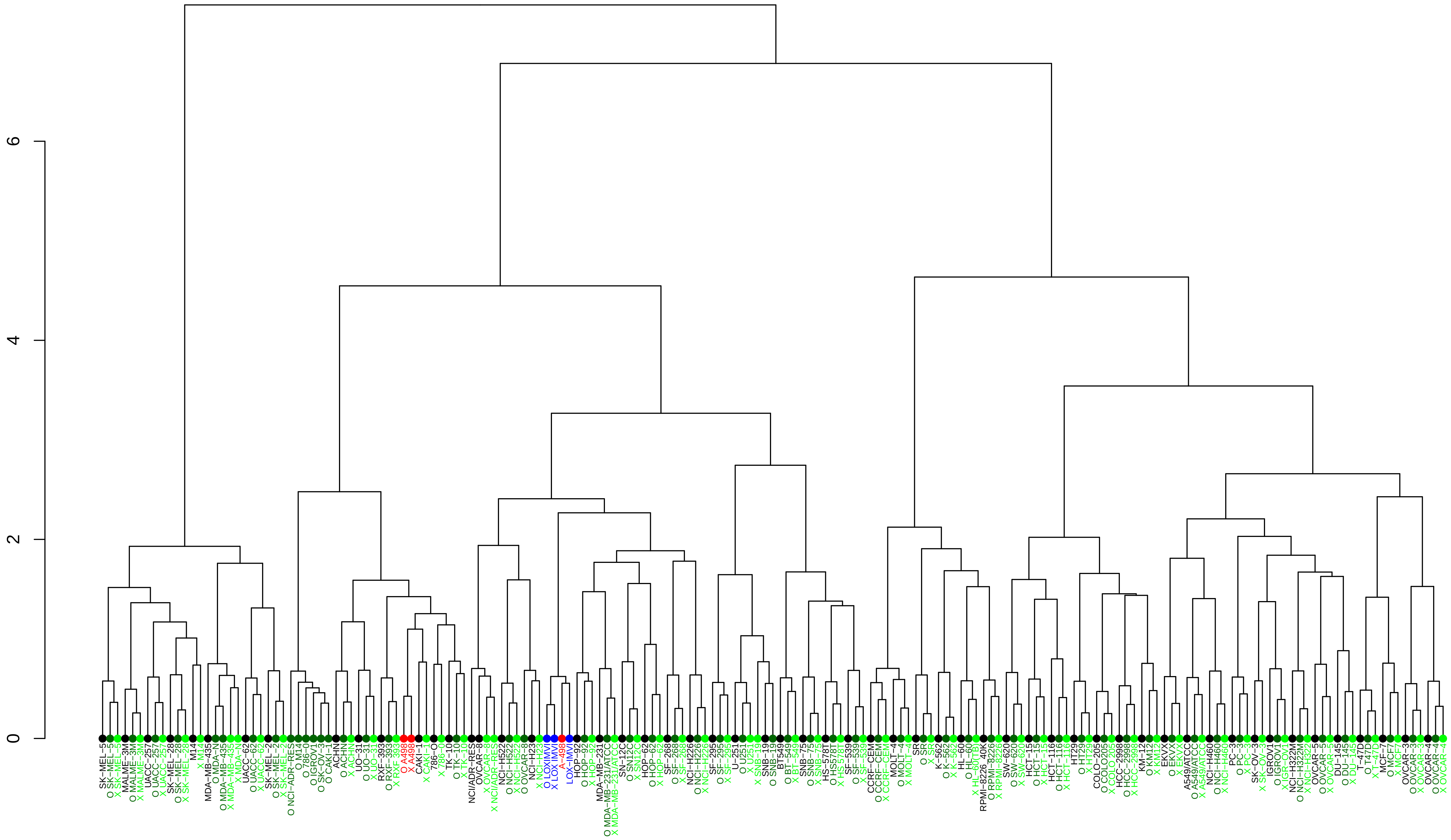

Figure S2. Clustering of the NCI60 from GSE7947 (black), GSE5949 (dark green, prefaced by an X) and GSE5720 (green, prefaced by an O), using Ward linkage and correlation. Cell lines duplicated between the three studies cluster together, with the exception of A498 (red) which clusters with LOXIMVI (blue) only in GSE7947.
